# The United States Health Service

**Published:** 1902-02

**Authors:** 


### THE UNITED STATES HEALTH SERVICE.

A bill has been introduced in Congress for the purpose of increasing the efficiency of the United States Marine Hospital Service, and changing its name to "The United States Health Service."

It is argued that the present name of the corps does not properly represent the character and scope of its work, and that when the field of its activities is still further enlarged the old name will be even less suitable than it is at present.

All officers and employes are to be retained and their pay is to remain as heretofore except in the case of the surgeon general, whose salary is to be increased to equal that received by the surgeon general in the army. This increase in the surgeon general's pay is simply an act of justice which should have been done many years ago. In dignity and responsibility his office is quite equal to those of the surgeon general's of the army and navy, and his duties as chief officer in charge of the administrative divisions of the United States Health Bureau, including marine hospitals and relief, domestic quarantine, foreign and insular quarantine, sanitary reports, statistics, scientific research, etc., are much more arduous than theirs.

